# Target foil rupture scenario and provision for handling different models of medical cyclotrons used in India

**DOI:** 10.4103/0971-6203.54851

**Published:** 2009

**Authors:** V. S. Shaiju, S. D. Sharma, Rajesh Kumar, B. Sarin

**Affiliations:** Radiological Physics & Advisory Division, Bhabha Atomic Research Centre, Mumbai - 400 094, India; 1 Shreeji Imaging & Diagnostic Pvt. Ltd, Navi Mumbai, India

**Keywords:** Emergency handling, Havar foil, medical cyclotron, radiation safety, target rupture

## Abstract

Medical cyclotron is a particle accelerator used in producing short lived radiotracers such as ^18^F, ^11^C, ^15^O, ^13^N etc. These radiotracers are labeled with suitable pharmaceuticals for use to gather information related to metabolic activity of the cell using Positron Emission Tomography (PET) scan. Target foil rupture is considered one of the major emergency situations during medical cyclotron operations because there is a potential of over exposure to the working personnel. Radiation protection survey of a self-shielded medical cyclotron installation was carried out during normal and emergency conditions. It is found that the induced activity in the target foil increases with its successive usages. As a case study, we have evaluated the emergency handling procedures of GE PETtrace-6 medical cyclotron. Recommendations have also been made to reduce personal exposure while handling the target foil rupture condition such as the use of L-Bench near the target area and participation of experienced personnel.

## Introduction

Medical cyclotron facilities are used to produce radiotracers required for Positron Emission Tomography (PET) scans. The PET scan provides information related to the metabolic activity of the cell which is more important in modern cancer treatment techniques.[1–2] In general, medical cyclotron accelerates negatively charged hydrogen ion. Medical cyclotron is designed in such a way that the induced radioactivity within the accelerator is minimal. Therefore, with proper safety procedures and precautions, the radiation exposure to the operating staff is almost negligible. Because of the high necessity and low half life of the cyclotron produced radiotracer, medical cyclotron installations are increasing in number in the country. These medical cyclotron installations should satisfy the norms and regulatory requirements of national competent authority (AERB), which ensures the safety of the installation and operation of medical cyclotron. Medical cyclotron operations have potential for accidents such as target foil rupture, which require emergency planning and procedures. In this study we have evaluated the target foil rupture scenario and handling procedures in GE PETtrace-6 medical cyclotron model.

## Materials and Methods

### Construction and Working Principle of Medical Cyclotron

**Figure 1:**
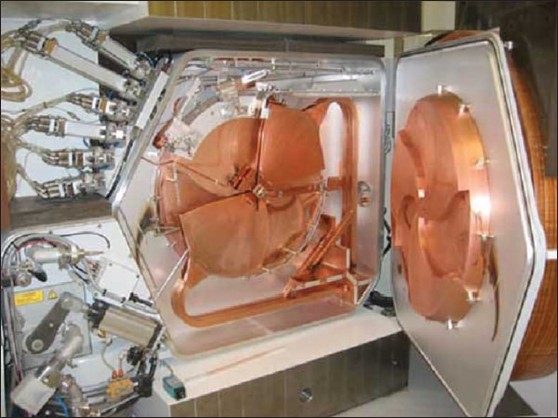
Dee's, ion source and target system of a medical cyclotron

Cyclotrons accelerate charged particles using a high-frequency alternating voltage. A perpendicular magnetic field causes the particles to spiral in a circular path so that they re-encounter the accelerating voltage many times. Figure 1 shows a medical cyclotron which consists of a pair of ‘D’-shaped electrodes, an ion source at the centre, a static magnetic system to keep the ion in a circular path, a stem to supply RF power to the Dee's, a stripping foil to change the polarity of the ion, and a target system. Negatively charged ions are extracted from the ion source using alternating high voltage and accelerate using RF power. Using a static magnetic field keeps these ions in a circular path in ‘Dee’ assembly. The beam extraction system consists of a stripper foil, which changes the ion polarity to positive and thus steering the ions to hit the target according to the target selection. Details of radio-nuclides commonly produced in the medical cyclotron are shown in the Table 1.

**Table 1:** Details of radio nuclides produced in a medical cyclotron

*Radio tracer*	*Half life (Min)*	*Target medium*	*Nuclear reaction*	*Beam energy*
^8^F	110	Enriched ^18^O water	^18^O(p, n)^18^F	16.5MeV
^11^C	20	N_2_	^14^N(p, α)^11^C	16.5MeV
^l5^O	2	N_2_	^14^N(d, n)^15^O	8.4MeV
^13^N	10	Distilled water	^16^O(p, α)^13^N	16.5MeV

### Radiation Safety Aspects

Radiation safety aspects of medical cyclotron installations include shielding adequacy, safety interlocks, negative pressure in cyclotron room, proper radiation monitoring instruments, uninterrupted power supply and generator back-up for full facility, etc. The self-shielded cyclotron model comprises of several stainless steel tanks filled with lead bricks and boronated water to shield against gamma and neutron radiations.[3] The first layer consists of lead placed close to the target to reduce gamma flux from the target area. The next layer boron loaded polyethylene captures neutrons. An additional layer of lead in tank captures the gammas created by slow neutrons. The boronated water-filled tanks stop the remaining neutrons. In the self-shielded cyclotron the beam cannot be initiated on target unless shield bricks are closed and safety interlocks actuated. The interlock system handles malfunctions and operational errors to protect personnel and equipment. The interlocks are designed so that when an interlock is tripped, the cyclotron operation stops and cannot be restarted until the interlock has been reset. Gamma area monitors and neutron monitors with audible alarm inside and outside the control room should be functional during the cyclotron operation. In case of any failure, the cyclotron controller stops the beam. The cyclotron can operate only if the ventilation system is operational and ventilation safety functions are to be tested prior to the facility startup.

### Ventilation System

A proper ventilation system ensures the dynamic containment of the cyclotron installation. The pressure difference provides this dynamic containment function. Contaminated air from the cyclotron system is passed through the HEPA (High-Efficiency Particulate Air) filters and then through the carbon filters which ensures the continuity of the containment and stored in Automatic Air Compression System (ACS). The contaminated air thus collected is released after 10 half lives decay through an exhaust. Exhaust air is also monitored by the ventilation duct radiation monitor. The radiation detector is installed inside the duct system to monitor the activity constantly irrespective whether beam is ON or OFF. The ducts of the ventilation system remain open during or after beam operation. Potentially contaminated air from the cyclotron is monitored to ensure exposure rates below country's permissible limit as per AERB's guidelines and is disseminated in the environment through a chimney on the roof of the building.

Ventilation is required for moisture condensation problems in the vault as well as the removal of corrosive ozone gas, ^15^O, ^13^N, etc. generated by high energy electrons and X-rays from the cyclotrons, which can cause risk to the personnel going inside the vault of a cyclotron. The combination of high energy electrons, X-rays, and ozone create a highly corrosive environment in the vault. AERB regulations specify that the ozone content in work areas must not exceed 0.1ppm by volume. Persons going inside the vault should wait several minutes after the high voltage has been shut off, before entering, to ensure that the ozone level has dropped below this value.

The ventilation safety functions have to be tested prior to the facility start-up. The ventilation power supply should be protected to avoid any ventilation loss during the cyclotron operation.

### Quality Control

To maintain quality control and system performance, major technical problems like run failure, radiation detector failure, power failure, chilled water supply pumps failure, vacuum failure, etc. should be avoided. Alarms and erroneous detector readings should be avoided by replacing old filters and adjusting the inlet and outlet air pressure of the duct system. The target should be kept clean, loaded, pressurized and bombarded properly.[1]

### Target System

**Figure 2:**
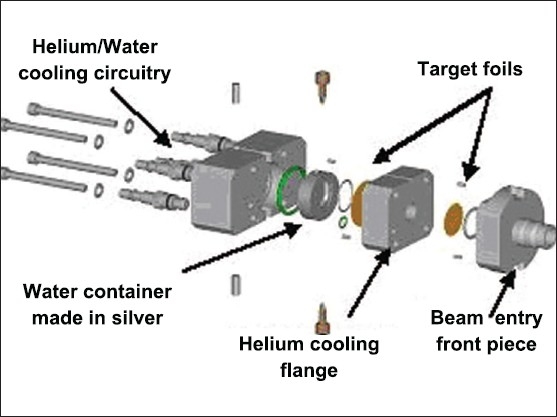
Target assembly of a medical cyclotron containing Havar foils

The beam enters the target chamber through a double-foil assembly. The target assembly consists of a front flange, helium cooling flange, target chamber and a rear flange as shown in Figure 2. Target chamber is made up of silver. The front flange supports the beam into the target chamber for irradiation. Two thin metal foil windows (Havar foils) are used to separate the target chamber from the cyclotron vacuum. Havar foil forms the entrance of proton beam into a highly pressurized ^18^O enriched water target used for the production of ^18^F. Havar is an alloy containing cobalt (42.5%), chromium (20%), nickel (13%), tungsten (2.8%), molybdenum (2%), manganese (1.6%), carbon (0.2%), beryllium (0.04%), and iron (balance).[4] This cobalt base alloy has no magnetic attraction and high melting point. In the target assembly, the front side and the rear side Havar foils have an approximate thickness of 50 *μ*m and 25 *μ*m respectively. High speed re-circulating helium gas is used for Havar foil cooling. Cooling water is also circulated from the rear side of the target assembly whenever the system selects the target.

### Cyclotron Emergencies

The important consideration at the time of medical cyclotron installations includes space requirement, shielding configuration, safety interlocks, proper ventilation, etc. According to various manufactures of medical cyclotron the major emergency events in the medical cyclotron facility are: (1) target pressure loss (target foil rupture); (2) radioactive material delivery to/from the cyclotron target is delayed for more than 10 minutes beyond the expected time, and (3) accidental radioactive material contamination or exposure.

### Target Foil Rupture Scenario

**Figure 3:**
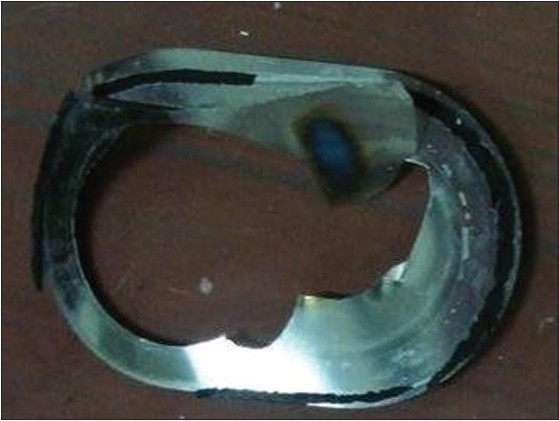
Ruptured havar foil of a medical cyclotron

Technical information related to the various medical cyclotrons used in India was scrutinized and it was found that the target rupture is one of the major emergencies during the cyclotron operation. Accordingly, almost similar emergency handling procedures have been suggested by the manufactures. However, we practically studied the emergencies during target rupture scenario in GE PETtrace-6 medical cyclotron on five occasions. It is mainly due to the failure in target cooling system. According to the vendors, the typical frequency of the target foil rupture is about once in 300 cyclotron runs. The ruptured Havar foil is shown in Figure 3. Table 2 shows the operational parameters of a self-shielded GE PETtrace-6 medical cyclotron.[3] All PETtrace cyclotrons can be configured to support dual ^18^F targets that may be irradiated individually or simultaneously so that if one target has ruptured foils then the irradiation can be switched to the other target with no emergency situation arising. However, the PETtrace-6 at Shreeji Imaging and Diagnostic Pvt. Ltd. is a unique one. The main source of elevated exposure rate during the target foil rupture is the induced activity produced in the Havar foils, when all the target activity has been pumped away or after a suitable cool down time. During cyclotron operation the Havar foils get exposed both to an intense primary beam of protons and to a large flux of secondary neutrons produced by the ^18^O (p, n) ^18^F reaction. There were twelve induced radionuclides, ^48^V, ^51^Cr, ^52^Mn, ^54^Mn, ^56^Co, ^57^Co, ^58^Co, ^60^Co, ^95m^Tc, ^96^Tc, ^183^Re, and ^184^Re in Havar foil. The induced activity depends on the target usage expressed in ‘μA.hr’. Initially the induced activity in the Havar foil is dominated by activities of ^56^Co and ^58^Co. However, the half life of these radio-nuclides is less and after a long time the dominant radio-nuclides will be induced ^60^Co. The exempt activity of ^60^Co prescribed in the International Basic Safety Standards (BSS) is 0.1MBq.[4] During foil rupture, it constitutes a solid radioactive waste whose handling, storage, and disposal must be done very carefully. From the aspect of radioactive waste management, the used Havar foil should be managed about 20 years to attain the exempt activity. The radiation monitoring is also needed for the above procedure.

**Table 2:** Operational parameters for proton beam for GE PETtrace-6

RF Frequency	27.2MHz
Beam current	60 μA
Nominal beam energy	16.5MeV
Extraction radius (Average)	0.32m
Dee Voltage	35KV
Vacuum (beam on)	4.10^−5^±2.10^−5^ mbar
Magnetic coil current	410Amps
Target pressure	400-450psi
‘He’ cooling pressure	45-55psi

### Emergency Planning Procedures

A fault on the beam or the target cooling system can cause a target foil rupture scenario in medical cyclotron.[5] In case of rupture of the window separating the target from the window cooling circuit the cyclotron controller stops the irradiation and immediately closes the extraction port valves and the containment is ensured by the second window. The helium-water mixture is eliminated after ^18^F decay. If both the windows are ruptured, the cyclotron controller will stop the irradiation and will immediately close the extraction valves. Fluorinated water will remain in the cyclotron and in the vacuum pump oil. The vacuum pump vents are connected to the building exhaust system, the HEPA filter in the ventilation will ensure a redundant safety barrier. Foil fragments lodged in the collimator assemblies may cause an electrical short circuit that may make the cyclotron difficult to operate after shutting down the cyclotron and determine the extent and severity of the emergency situation. Before entering the vault wear protective clothing, TLD dosimeter, pocket dosimeter for instantaneous dose measurements and a calibrated survey meter to identify areas with elevated exposure rates. Measure the radiation level inside the cyclotron vault and if safe, open the shielding door of cyclotron and again measure the radiation level on the target surface. If the radiation level is high, it is advisable to wait until an acceptable limit which may depend up on the number of personnel involved and the time required to remove the target. If the radiation level is safe to handle, remove the target from the cyclotron and keep it behind enough lead shielding in an earth pit with proper labels and warning symbols. Measure the radiation level outside the pit. Removal of foil fragments from the cyclotron can be a time-consuming process. These fragments should be removed. Then open the ‘Dee’ assembly and check for ruptured foil pieces and remove the foils using long forceps. Clean the tank with cleaning liquid and tissue paper, keeping in mind there may be a chance of contamination if the irradiated O-18 water leaked into the tank. Take the target behind the L-bench for maintenance only when the radiation level is safe to handle.

**Figure 4:**
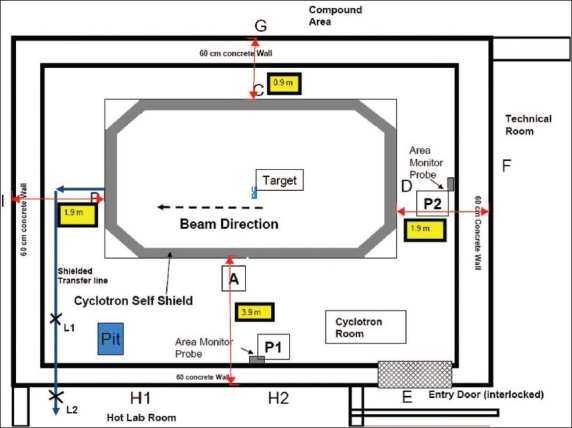
Layout of a self-shielded medical cyclotron installation. A, B, C, D, E, F, G, I, P1 and P2 represents positions where radiation survey was carried out

The radiation measurements are taken using a RM 703A (Nucleonix) survey meter before, during and after the normal cyclotron beam operation. The readings are tabulated in Table 3. RM 703A survey meter is a G.M. based detector to measure X and γ-radiations. Its energy response is within ±20% from photon energy 60 keV to 1.25 MeV. The neutron dose is also tabulated using a DigiPig (Wedholm) neutron monitor with the same condition. This detector measures the neutron dose rate in the unit of ‘mSv/hr’ in the energy range from thermal neutron to 17 MeV. This instrument is essentially independent of direction of the source and has a low sensitivity to γ-radiation. The measurements locations are indicated in the lay-out of GE PETtrace-6 cyclotron as shown in Figure 4. The corresponding measured data is tabulated in Table 3. In the target foil rupture scenario, we have measured the surface doses before and after removing the ruptured foil. Five such scenarios were studied and tabulated in Table 4. These measurements were taken after the complete decay of the residual target activity.

**Table 3:** Radiation survey measurements in cyclotron vault (Operational beam current 60μA)

*Survey Meter*	*RM 703A (Nucleonix)*			*DigiPig (Wedholm)*		
		
*Position labeled in the lay-out*	*Gamma (μSv/h)*			*Neutron (μSv/h)*		
	
	*Before Irradiation*	*During Irradiation*	*After Irradiation*	*Before Irradiation*	*During Irradiation*	*After Irradiation*
A	0.14	80.7	0.175	0	6	0
B	0.13	18.59	0.16	0	3	0
C	0.114	78	0.16	0	1	0
D	0.09	14	0.17	0	0	0
E	0.07	1.92	0.14	0	0	0
F	0.07	0.184	0.09	0	0	0
G	0.09	0.09	0.09	0	0	0
I	0.09	0.09	0.09	0	0	0
P1	0.09	10.4	0.09	0	0	0
P2	0.09	3.16	0.09	0	0	0

**Table 4:** Dose survey on the surface of the target before and after removal of ruptured foil (Measured after complete decay of the residual target activity)

*Target Usage (μA.h)*	*Dose measured on surface of Target with intact ruptured foil (mSv/h)*	*Dose measured on surface of Target after removing the ruptured foil (mSv/h)*
6000	62	0.140
3500	37	0.070
3000	30	0.060
1700	25	0.035
3500	35	0.080

## Discussion

The radiation survey of the cyclotron installation shows comparatively higher radiation level at positions A and C, which is on the surface of the self-shielding tank perpendicular to the bean direction. The GE PETtrace was operated with 60 *μ*A beam intensity and the measured values are shown in Table 3. However, readings of these locations show a similar radiation levels during the foil rupture scenario. The gamma zone monitor P1 shows a higher radiation level during the beam on due to the streaming radiation from the self-shield of the cyclotron.

From Table 4, it is clear that the dose measured on the surface of the target with intact of ruptured foil is directly proportional to the target usage. The measurements were taken after the complete decay of residual activity of the target. The data corresponds to five such scenarios of target failure. As the target usage increases the induced activity within the target foil will also increases. In similar target usage conditions the variations in readings might be due to the different amount of scattered Havar foil pieces in to the vacuum chamber.

Considering the induced activity buildup in the cyclotron assembly, it is recommended by manufactures to wait at least 24 hours to open the cyclotron assembly to reduce the activity of short lived radio-nuclides produced in the cyclotron. There should be a work practice protocol for the safe removal of the ruptured foils. Otherwise it would lead to over exposure to the personnel involved. It is also advisable to rotate the man power during the handling procedure in order to reduce the individual dose. However, removal of Havar foil needs to be done by experienced workers. Even though the events of emergencies are rare, mock trial of the emergency handling procedure is also advisable. Both experience and skill play an important role in the emergency handling procedure to minimize exposure to the personnel.

In the current working practice, the ruptured target is taken out manually to keep into a lead container which is located outside the self-shield. However, it is recommended to adopt a methodology to spend minimum time with the ruptured target during the handling procedure. Here we suggest that taking the trolley attached L-Bench inside the cyclotron self-shield reduces time and hence the dose to the personnel.

## Conclusion

Target foil rupture is one of the major emergencies which can lead to exposure to the personnel involved with cyclotron operation. This study evaluates the importance of the emergency handling procedures to minimize exposure to the radiation worker. Technical reports on emergency planning procedures for the target rupture scenario of different cyclotron manufactures were scrutinized and found similar. Radiation survey of the target rupture scenario was studied on GE PETtrace-6 medical cyclotron at Shreeji Imaging and Diagnostic Pvt. Ltd. It is also found that the induced activity in the target foil increases with its successive usages. Personnel exposure during these procedures can be reduced by introducing the access of L-Bench nearer to the cyclotron target and with the experience of qualified personnel participation.
